# Development of an exoglycosidase plate-based assay for detecting α1-3,4 fucosylation biomarker in individuals with HNF1A-MODY

**DOI:** 10.1093/glycob/cwab107

**Published:** 2021-10-25

**Authors:** Daniel Demus, Paulina A Urbanowicz, Richard A Gardner, Haiyang Wu, Agata Juszczak, Tamara Štambuk, Edita Pape Medvidović, Katharine R Owen, Olga Gornik, Nathalie Juge, Daniel I R Spencer

**Affiliations:** Ludger Ltd., Culham Science Centre, Abingdon, OX14 3EB, United Kingdom; Center for Proteomics and Metabolomics, Leiden University Medical Center, Albinusdreef 2, 2333 ZA, Leiden, The Netherlands; Ludger Ltd., Culham Science Centre, Abingdon, OX14 3EB, United Kingdom; Ludger Ltd., Culham Science Centre, Abingdon, OX14 3EB, United Kingdom; The Gut Microbes and Health Institute Strategic Programme, Quadram Institute Bioscience, Norwich Research Park, Norwich, NR4 7UQ, United Kingdom; Oxford Centre for Diabetes, Endocrinology and Metabolism, University of Oxford, Oxford, OX3 7LE, United Kingdom; Genos Glycoscience Research Laboratory, Borongajska cesta 83h, 10000, Zagreb, Croatia; Faculty of Pharmacy and Biochemistry, University of Zagreb, Ante Kovačića 1, 10000 Zagreb, Croatia; Vuk Vrhovac University Clinic for Diabetes, Endocrinology and Metabolic Diseases, Merkur University Hospital, Zagreb University School of Medicine, Dugi dol 4A, 10000, Zagreb, Croatia; Oxford Centre for Diabetes, Endocrinology and Metabolism, University of Oxford, Oxford, OX3 7LE, United Kingdom; NIHR Oxford Biomedical Research Centre, Oxford Hospitals NHS Foundation Trust, Oxford, OX3 9DU, United Kingdom; Faculty of Pharmacy and Biochemistry, University of Zagreb, Ante Kovačića 1, 10000 Zagreb, Croatia; The Gut Microbes and Health Institute Strategic Programme, Quadram Institute Bioscience, Norwich Research Park, Norwich, NR4 7UQ, United Kingdom; Ludger Ltd., Culham Science Centre, Abingdon, OX14 3EB, United Kingdom

**Keywords:** biomarker, diabetes, fucosylation, glycans, hnf1a-mody

## Abstract

Maturity-onset diabetes of the young due to hepatocyte nuclear factor-1 alpha variants (HNF1A-MODY) causes monogenic diabetes. Individuals carrying damaging variants in *HNF1A* show decreased levels of α1-3,4 fucosylation, as demonstrated on antennary fucosylation of blood plasma *N*-glycans. The excellent diagnostic performance of this glycan biomarker in blood plasma *N*-glycans of individuals with HNF1A-MODY has been demonstrated using liquid chromatography methods. Here, we have developed a high-throughput exoglycosidase plate-based assay to measure α1-3,4 fucosylation levels in blood plasma samples. The assay has been optimized and its validity tested using 1000 clinical samples from a cohort of individuals with young-adult onset diabetes including cases with HNF1A-MODY. The α1-3,4 fucosylation levels in blood plasma showed a good differentiating power in identifying cases with damaging *HNF1A* variants, as demonstrated by receiver operating characteristic curve analysis with the AUC values of 0.87 and 0.95. This study supports future development of a simple diagnostic test to measure this glycan biomarker for application in a clinical setting.

## Introduction

Glycosylation is a co-/posttranslational modification of proteins which is important for protein structure, stability and function, and influences most biological processes including cell signaling. There are two types of glycosylation: *N*-glycosylation where glycans are attached to a protein via a glycosidic bond to asparagine and *O*-glycosylation where the glycosidic bond links glycans to either serine or threonine (Varki 2017). Advances in analytical technologies have helped to further understand the role of glycosylation in human health and disease. Alterations in glycosylation patterns occur in certain pathological conditions such as cancers (Hu et al. 2019), autoimmune diseases (Seeling et al. 2017) and in response to lifestyle changes (Knežević et al. 2010). Glycosylation features, such as sialylation, fucosylation and galactosylation, have been explored in different diseases as potential biomarkers (Hu et al. 2019) for early diagnosis (Gudelj et al. 2018) or for monitoring the effectiveness of treatments (Ercan et al. 2010; Lundström et al. 2017). The measurement of glycosylation features may also be applied to estimate and track the biological age of healthy individuals (Gudelj et al. 2015).

Maturity onset diabetes of the young due to damaging alleles in hepatocyte nuclear factor-1 alpha (HNF1A-MODY) is a rare type of diabetes caused by an autosomal-dominant mutation in the single gene, *HNF1A*, which is involved in regulating β-cell development and insulin secretion (Cardenas-Diaz et al. 2019). HNF1A-MODY is characterized by defects in insulin secretion and onset of hyperglycemia in the 2nd-4th decade of life (Urakami 2019). A correct diagnosis is a key factor for optimal disease management, as early stages of HNF1A-MODY can be effectively controlled with orally administered sulfonylureas (Pearson et al. 2003). Diagnosis of HNF1A-MODY is challenging as it often depends on the awareness of physicians and the availability of relatively expensive genetic testing for confirming the presence of pathogenic variants in the *HNF1A* gene. Moreover, HNF1A-MODY shares clinical features with other types of diabetes which is estimated to lead to misdiagnosis in ~80% of cases in the United Kingdom (Shields et al. 2010). A MODY probability calculator has been developed to help physicians detect those at higher risk of MODY (Shields et al. 2012). Moreover, due to the autosomal dominant inheritance pattern of HNF1A-MODY, a correct diagnosis helps identify other family members affected by this disorder (Urakami 2019).

Certain variants in the *HNF1A* gene result in altered glycosylation patterns associated with HNF1A-MODY. Genome-wide association studies (GWAS) show that damaging loss-of-function variants in the *HNF1A* gene lead to downregulation of fucosyltransferases that perform the α1-3 and α1-4 fucosylation of glycans (Lauc et al. 2010). Further studies proposed that decreased α1-3 and α1-4 fucosylation of *N*-glycans (antennary fucosylation) from blood plasma proteins can be used as a differentiating biomarker for HNF1A-MODY (Thanabalasingham et al. 2013). The use of antennary fucosylation as a biomarker of HNF1A-MODYwas previously demonstrated (Thanabalasingham et al. 2013; Juszczak et al. 2019) and we recently showed the excellent interlaboratory performance of the *N*-glycan biomarker using liquid chromatography (LC) methods (Demus et al. 2021). Nevertheless, the applicability of LC-based methods in clinical practice is limited due to their low robustness and equipment-related costs; therefore, development of a simpler analytical approach to test for this well-studied biomarker is warranted.

Here, we have developed an exoglycosidase plate-based assay which enables high-throughput measurements of levels of fucose residues that are attached via α1-3 and α1-4 linkages to glycoconjugates present in blood plasma. The assay has been optimized and its performance validated on 1000 clinical blood plasma samples from individuals with young-adult onset diabetes, including groups of individuals with different variants in the *HNF1A* gene. The differentiating power of α1-3,4 fucosylation levels is comparatively discussed with the previously published results testing antennary fucosylated *N*-glycans as a biomarker for HNF1A-MODY using LC methods.

## Results

### Development of the plate assay for measurements of α1-3,4 fucosylation levels in plasma samples

**Fig. 1:**
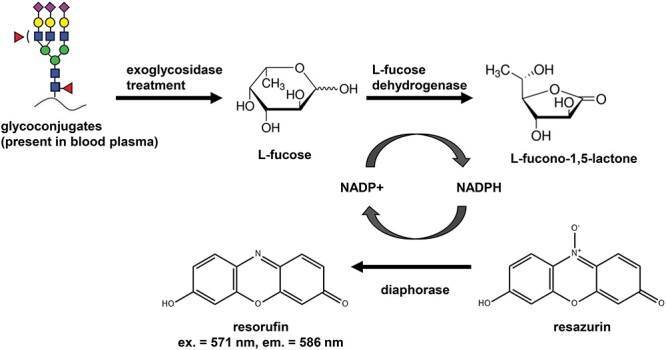
Schematic of the enzymatic redox reaction resulting in the formation of fluorescent product resorufin that forms the detection mechanism of the exoglycosidase plate-based assay. Resorufin measured at an excitation (ex.) wavelength of 571 nm and an emission (em.) wavelength of 586 nm. [NAPD+: nicotinamide adenine dinucleotide phosphate (oxidized); NADPH: nicotinamide adenine dinucleotide phosphate (reduced)]. This figure is available in black and white in print and in colour at *Glycobiology* online.

The exoglycosidase plate-based assay developed in this work is based on the use of a novel α1-3,4 specific fucosidase (E1_10125), which is characterized by the ability to remove fucose residues from glycoconjugates present in blood plasma (Wu et al. 2020; Demus et al. 2021). Importantly, E1_10125 is capable of removing antennary fucose residues from *N*-glycans terminated with sialic acids, which is an advantage over commercially available α1-3,4-specific fucosidases. The released L-fucose residues are then subjected to an enzymatic redox reaction to produce a fluorescence signal which is directly proportional to the amount of released L-fucose residues. The released L-fucose monosaccharides are oxidized to L-fucono-1,5-lactone by a L-fucose dehydrogenase which in turn reduces NADP+ to NADPH. The NADPH is then oxidized by a diaphorase which leads to the reduction of resazurin and the formation of the fluorescent product resorufin in molar proportions stoichiometric to the released L-fucose monosaccharides (Figure 1). The fluorescence signal reaches maximum levels following 3 h incubation and provides the best signal to background ratios. Each plasma sample was either treated with the exoglycosidase or untreated and processed in the same manner throughout the different steps of the plate-based assay. The fluorescence signal of the untreated sample was subtracted from the fluorescence signal of the exoglycosidase-treated sample to exclude fluorescence background and possible interferences coming from the sample matrix. The amount of fucose in the plasma samples was then calculated based on the fucose standard curve. No fluorescence signal was generated by the E1_10125 alone, which might have influenced fucosylation level readouts and the consistency of measurements (data not shown). In this type of assay based on fluorescence measurements, readouts of low concentrations of monosaccharides might be limited by the fluorescence background signal. To be accurate, the fluorescence signal of the sample (following background subtraction) should always be greater than 0 (Table S1).

To determine the optimal concentration of E1_10125 during the development of the assay, 2-, 4-, 8- and 16-μM concentrations of the enzyme were tested on 10-μL pooled blood plasma samples. The results showed similar levels of released fucose residues irrespective of the enzyme concentration (Figure S1A). Since protein concentration may vary between patient plasma samples (Leeman et al. 2018), we ensured that the final concentration of E1_10125 provides maximum and consistent release of fucose regardless of protein content in plasma samples, as demonstrated on a range of pooled plasma volumes (6, 8, 10, 12 and 14 μL) (Figure S1B). Based on these results, the optimal final concentration was determined to be 3 μM. Of note, in the development stage, a broader range of plasma volumes (2–18 μL) was tested. However, plasma volumes ≥16 μL led to the formation of a larger protein pellet after centrifugation, which disrupted aspiration and transfer of fucose-containing supernatants on the robotic platform, resulting in the need for manual intervention. This issue was not observed during a validation and the sample cohort analysis when using 10 μL of patients’ plasma samples, which indicates that a range of 6–14 μL mimics the protein content variation sufficiently. Furthermore, we demonstrated that E1_10125 fucosidase at 3 μM showed high efficiency (∼75%) in removing antennary fucose residues from intact glycoproteins present in blood plasma samples under assay conditions. This was directly compared to the efficiency of E1_10125 when applied to released *N*-glycans and showed that E1_10125 does not require released glycan substrates (Figure S2).

### Performance of the assay in analysing α1-3,4 fucosylation levels as a biomarker for HNF1A-MODY

Having optimized the exoglycosidase plate-based assay, 1000 blood plasma samples from the HNF1A-MODY cohort were analyzed to assess α1-3,4 fucosylation levels and evaluate their diagnostic performance for the identification of cases with diabetes carrying damaging variants in the *HNF1A* gene.

Based on systematic and functional assessment of rare *HNF1A* alleles, which had been performed previously (Juszczak et al. 2019), the 947 participants were grouped into four *HNF1A* variant types: (likely) damaging (*n* = 18), (likely) benign (*n* = 8), variants of unknown significance (VUS, *n* = 5) and no *HNF1A* rare variants (*n* = 916). Clinical characteristics of study participants are summarized in Table I.

**Table I:** Clinical characteristics of study participants

	(Likely) damaging allele, *n* = 18 cases	(Likely) benign, *n* = 8 cases	VUS, *n* = 5 cases	No rare HNF1A allele variant, *n* = 916 cases
Sex, male (*n*)	5	4	2	529
Age at recruitment (years)	40 ± 17	48 ± 11	54 ± 14	47 ± 11
Age at diagnosis (years)	26 ± 9	36 ± 6	37 ± 10	35 ± 7
Diabetes duration (years)	13 ± 12	9 ± 7	17 ± 11	12 ± 10
BMI (kg/m^2^)	26 ± 5	35 ± 5	28 ± 6	31 ± 7
hsCRP (mg/L)	0.8 ± 1.3	5.2 ± 5.7	4.0 ± 6.3	6.2 ± 29.7
HbA1c (%)	7.7 ± 1.7	8.8 ± 2.1	6.7 ± 1.3	7.9 ± 2.7
C-peptide (nmol/L)	0.42 ± 0.21	0.81 ± 0.62	0.60 ± 0.57	5.11 ± 71.47
Total cholesterol (mmol/L)	4.75 ± 1.09	5.07 ± 0.88	4.74 ± 1.21	4.69 ± 1.23
HDL (mmol/L)	1.4 ± 0.3	1.1 ± 0.2	1.3 ± 0.4	1.3 ± 3.4
Triglycerides (mmol/L)	1.2 ± 0.5	2.0 ± 1.0	1.1 ± 0.3	2.0 ± 1.7

Mean values are presented for each group (±SD).

VUS: variants of unknown significance.

From the 1000 samples tested, 947 showed a background to fluorescence signal >0 and blood plasma α1-3,4 fucosylation levels from these samples were subsequently subjected to statistical analyses.

**Fig. 2:**
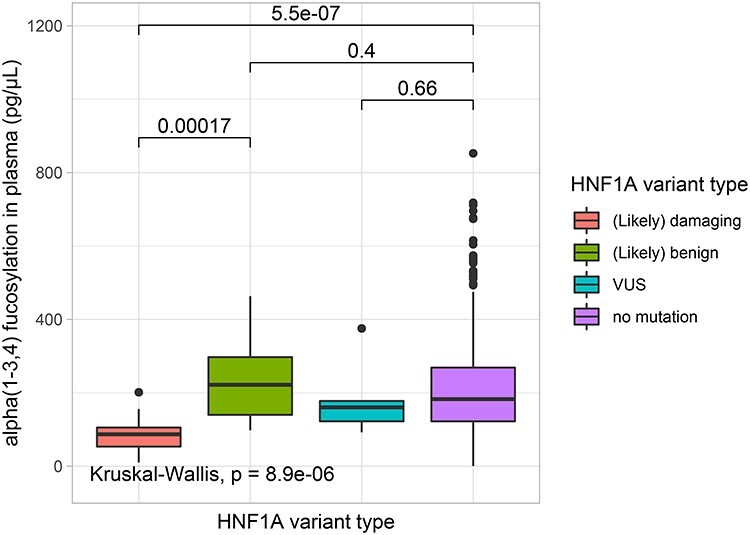
Box plots presenting differences in α1-3,4 fucosylation levels in blood plasma (pg/μL) measured using the exoglycosidase plate-based assay for groups of patients with different *HNF1A* variant groups. Each box represents 25th–75th percentile, the median is marked by a vertical line, whiskers indicate values that are within 1.5 × IQR of the hinge. Outliers are displayed as black filled circles. The lines and numbers above the box plots indicate the *P*-value when comparing two categories using The Wilcoxon–Mann–Whitney test. The analysis with *P* ≤ 0.05 is considered statistically significant. This figure is available in black and white in print and in colour at *Glycobiology* online.

Box plot analysis was used to evaluate differences in α1-3,4 fucosylation levels between the four *HNF1A* variant groups. The analysis showed significant differences (*P* ≤ 0.05) in α1-3,4 fucosylation levels between groups of (likely) damaging vs. (likely) benign and (likely) damaging vs. cases without rare *HNF1A* variants (Figure 2). No significant difference in α1-3,4 fucosylation levels was observed between (likely) benign and a group of cases without rare *HNF1A* variant groups. Data outliers were identified within each group, 1 case within (likely) damaging group and 24 cases within cases without *HNF1A* variants and removed prior to the further analyses (Kwak and Kim 2017). The average fucose content in blood plasma for each defined group was as follows: 79.26 ± 35.63 pg/μL for (likely) damaging, 241.01 ± 125.41 pg/μL for (likely) benign, 185.71 ± 111.15 pg/μL for VUS and 193.60 ± 99.58 pg/μL for cases without rare *HNF1A* variants.

**Fig. 3:**
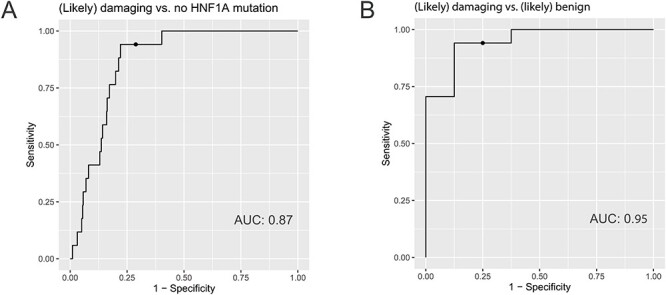
ROC curves illustrating the performance of α1-3,4 fucosylation levels in differentiating cases with (likely) damaging vs. no *HNF1A* variants (A) and (likely) damaging vs. (likely) benign variants in the *HNF1A* gene (B). The AUC values are displayed for each ROC curve. The optimal cut-off points are displayed as a dot on the precision recall curve for each ROC curve. This figure is available in black and white in print and in colour at *Glycobiology* online.

Next, receiver operating characteristics (ROC) curves were used to estimate the differentiating power of α1-3,4 fucosylation levels to identify patients with HNF1A-MODY carrying damaging variants in the *HNF1A* gene. The ROC analysis showed that α1-3,4 fucosylation levels provide good differentiating power between a group of (likely) damaging cases and the group of individuals without rare *HNF1A* variant, as determined by the measurement of the area under the curve (AUC) that was found to be 0.87 with 94% sensitivity, 71% specificity at the cut-off of 129.43 pg/μL (Figure 3A). Additionally, the diagnostic performance of α1-3,4 fucosylation levels was tested for (likely) damaging vs. (likely) benign variant groups giving an AUC of 0.95 with 94% sensitivity, 75% specificity at the cut-off of 137.59 pg/μL (Figure 3B). By applying α1-3,4 fucosylation levels as a biomarker for HNF1A-MODY, two cases from the VUS group would have been classified as carrying damaging variants.

### Intra- and interassay variations for measurements of α1-3,4 fucosylation levels in blood plasma samples using the exoglycosidase plate-based assay

In order to evaluate the effectiveness and robustness of the exoglycosidase plate-based assay, 72 plasma standard samples were distributed over 12 assay plates measured on different days. The results showed good repeatability and precision for measurements of α1-3,4 fucosylation levels in blood plasma samples with CVs of 9% for average intraplate variation and 10% for interplate variation (Table II).

**Table II:** Intra- and interassay variations for measurements of α1-3,4 fucosylation levels in blood plasma samples by the exoglycosidase plate-based assay. Intra- and interassay variations are described by CV values that were calculated based on the amount of released fucose for human blood plasma standards (*n* = 6) distributed over 12 assay plates

Intraplate variation			
Plate	Average fucose level in plasma (pg/μL), *n* = 6	SD	CV
1	235.65	13.11	6%
2	213.89	55.97	26%
3	224.09	30.18	13%
4	200.53	10.62	5%
5	250.55	6.62	3%
6	219.50	17.17	8%
7	242.71	10.95	5%
8	213.80	27.16	13%
9	256.58	7.85	3%
10	230.28	8.42	4%
11	175.92	19.79	11%
12	220.35	22.09	10%
Average intraplate			9%
Interplate variation	223.65	22.14	10%

CV: coefficient of variation.

### Associations between α1-3,4 fucosylation levels, age, sex and clinical markers

We investigated associations between α1-3,4 fucosylation levels and either sex or age within individuals with diabetes without rare variants in the *HNF1A* gene. A significant association between α1-3,4 fucosylation levels and sex (regression coefficient *β* = 0.25, *ρ* = 0.0005) but not age was observed. Average α1-3,4 fucosylation levels were found to be 180.9 ± 97.8 pg/μL in females (*n* = 362) and 204.2 ± 100.2 pg/μL in males (*n* = 510).

Previously, we reported significant correlations between C-reactive protein (CRP) levels and antennary fucosylation levels, which were measured using antennary fucosylated *N*-glycan traits by an LC-based method (Demus et al. 2021). Here, we tested α1-3,4 fucosylation levels against a panel of inflammatory and metabolic markers (Table I). We found a weak correlation between α1-3,4 fucosylation and CRP levels (correlation coefficient *r* = 0.29, *P* < 2.2 × 10^−16^) within the unselected sample cohort of young-adult onset diabetes (Figure S3A). Another weak correlation was found for α1-3,4 fucosylation levels and body mass index (BMI) with a correlation coefficient *r* = 0.17, *P* < 1.2 × 10^–6^ (Figure S3B) in the same cohort.

**Fig. 4:**
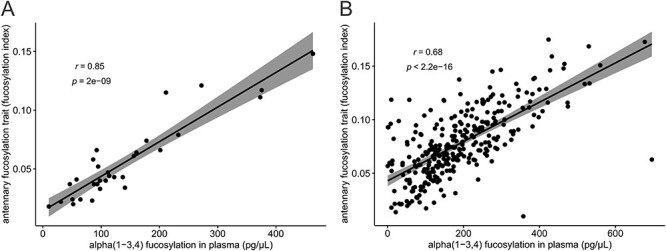
Correlations between *N*-glycan antennary fucosylation levels measured as indexes by LC method in the previous study (Demus et al. 2021) and α1-3,4 fucosylation levels measured by the exoglycosidase plate-based assay for 31 HNF1A-MODY positive cases (A), and 320 diabetes cases including 31 HNF1A-MODY positive and 289 negative cases (B). This figure is available in black and white in print and in colour at *Glycobiology* online.

We next compared the fucosylation indexes previously measured using an LC-based method (Demus et al. 2021), which reflect the levels of antennary fucosylation in the full plasma *N*-glycome, with the α1-3,4 fucosylation levels in blood plasma samples measured by the exoglycosidase plate-based assay. The analysis was carried out for 31 cases that had been diagnosed with variants in the *HNF1A* gene, which included groups of (likely) damaging, (likely) benign variants and VUS. The Spearman’s correlation analysis showed a strong correlation between the fucosylation indexes and α1-3,4 fucosylation levels in blood plasma samples with a correlation coefficient *r* = 0.85 (Figure 4A). When considering all previously analyzed 320 diabetes cases with and without variants in the *HNF1A* gene, a significant but weaker correlation with the correlation coefficient *r* = 0.68 was obtained (Figure 4B).

## Discussion

Diagnosis of HNF1A-MODY is challenging as current clinical criteria to guide genetic screening for MODY have been proven insensitive (Shields et al. 2017) and increasingly nonspecific (Misra and Owen 2018). Overlapping of clinical features of MODY with other types of diabetes, poor access and the cost of genetic testing often leads to misdiagnosis of MODY patients and incorrect disease management (Misra and Owen 2018). Since the GWAS study from 2010 revealed that damaging variants in the *HNF1A* gene are associated with decreased levels of antennary fucosylated *N*-glycans (Lauc et al. 2010), this potential glycan biomarker for HNF1A-MODY has been studied using a combination of fluorescently labeled glycans and LC separation techniques. Its diagnostic accuracy has been tested on large cohorts of cases with diabetes carrying different variants in the *HNF1A* gene. Individuals with damaging alleles in the *HNF1A* gene are known to have malfunctioning molecular mechanisms in which HNF1α factor is involved. The HNF1α is a master regulator of several genes, among others, genes controlling β-cell function and growth (Cardenas-Diaz et al. 2019) and genes encoding fucosyltrasferases (Lauc et al. 2010). Therefore, individuals that carry damaging variants in the *HNF1A* gene represent decreased levels of α1-3,4 fucosylation. The excellent differentiating performance of blood plasma antennary fucosylated *N*-glycans as biomarkers for HNF1A-MODY with the AUCs of ~0.90 has been reported (Thanabalasingham et al. 2013; Juszczak et al. 2019; Demus et al. 2021). By applying those glycan biomarkers, it was possible to identify rare cases with damaging variants in the *HNF1A* gene. However, this analytical method has not been translated into a widely available diagnostic tool.

As a response to the current demand for a simple and effective diagnostic tool for the detection of this well-studied *N*-glycan biomarker for HNF1A-MODY, we have developed an exoglycosidase plate-based assay with a fluorescence signal as the output to measure α1-3,4 fucosylation levels in blood plasma samples. This assay employs a novel α1-3,4-specific fucosidase (E_10125), which is capable of releasing fucose residues from glycoconjugates present in blood plasma samples (Wu et al. 2020). This fucosidase works efficiently on *N*-glycan structures terminated with sialic acids and shows high efficiency (∼75%) in removing antennary fucose residues from intact glycoproteins present in plasma samples. This advantage has been exploited in this study. The remaining portion (∼25%) of undigested fucose from *N*-glycans, when applying E1_10125 fucosidase to denatured plasma samples, might be due to the inaccessibility of fucose residue substrates to the enzyme, such as in this case where the glycans remain attached to the proteins. Nevertheless, the plate-based assay targets α1-3,4-linked fucose from all glycoconjugates (*N*- and *O*-glycosylated proteins and glycolipids) present in plasma samples and, as demonstrated by the consistency and excellent linearity of released fucose levels, the undigested portion of *N*-glycan fucose residues does not interfere with the results.

The validity of the assay was tested on 1000 blood plasma samples from a diabetes cohort, which included diagnosed cases with and without rare variants in the *HNF1A* gene. The ROC analysis showed that α1-3,4 fucosylation levels assessed from blood plasma samples by the exoglycosidase plate-based assay provide very good discriminatory power to identify cases with damaging variants in the *HNF1A* gene, as tested against a group of cases with benign (AUC = 0.95) and no rare variants (AUC = 0.87) in the *HNF1A* gene. The results for cases with damaging vs. no rare variants in *HNF1A* are similar to the AUCs of 0.90 for two single glycans structures reported using a LC-based method from the same sample cohort (Juszczak et al. 2019). We previously determined an antennary fucosylation trait, which reflects overall changes in antennary fucosylation in blood plasma *N*-glycome (Demus et al. 2021). Here, *N*-glycan antennary fucosylation levels measured as indexes by applying this derived antennary fucosylation trait were correlated with α1-3,4 fucosylation levels of blood plasma glycoconjugates measured by the exoglycosidase plate-based assay for 31 HNF1A-MODY cases and all 320 cases with young-adult onset nonautoimmune diabetes, separately. Variations of α1-3,4-linked fucose levels in glycoconjugates other than *N*-glycans present in blood plasma, which were not measured using the LC-based method, contribute to the plate-based assay signal and may account for the lower than the expected Spearman’s correlation coefficients of 0.85 and 0.68, respectively. Nevertheless, the correlation analysis together with results obtained for the intra- and interplate variations from the current cohort study demonstrate high repeatability of measurements and the validity of the exoglycosidase plate-based assay.

The performance of a biomarker based on α1-3,4 fucosylation might be influenced by inflammatory events that affect glycosylation profiles (Ogawa et al. 2020; Demus et al. 2021). A large proportion (∼50%) of *N*-glycans containing α1-3,4 fucosylation derive from acute phase proteins expressed during inflammation (Clerc et al. 2016), and here, we have confirmed that α1-3,4 fucosylation levels weakly correlate with CRP levels (correlation coefficient *r* = 0.29) that are indicative of inflammation as well as a nonalcoholic fatty liver disease related to participants BMI (Lee et al. 2017). For the latter, overexpression of fucosyltransferase FUT6 has been reported, which might lead to altered expression of other fucosyltransferases due to their interconnection with the HNF1α transcription factor and the availability of the guanosine diphosphate-fucose (GDP-fucose) donor substrate (Becker and Lowe 2003; Lauc et al. 2010). Furthermore, the weak correlation between α1-3,4 fucosylation levels and BMI (correlation coefficient *r* = 0.17), which has also been observed within the current sample cohort, might be explained by the obesity-linked proinflammatory state, and secretion of inflammatory mediators as well as CRP (Ellulu et al. 2016). In addition, we found that the α1-3,4 fucosylation levels were associated with sex, but not age, with higher α1-3,4 fucosylation levels in male individuals, consistent with previous findings reporting that fucosylation is gender dependent (Ding et al. 2011). Altogether, these data indicate that different cut-off values for α1-3,4 fucosylation levels will need to be applied to men and women in a real diagnostic setting.

CRP, which is already widely used in clinical testing, has been evaluated previously as a biomarker for HNF1A-MODY and provided a good diagnostic performance with an AUC of 0.83 (88% sensitivity and 69% specificity at the cut-off of 0.81 mg/L), although worse than the performance of antennary fucosylated *N*-glycan and α1-3,4 fucosylation biomarkers (Juszczak et al. 2019). In addition, the application of CRP as a specific biomarker for HNF1A-MODY is limited as CRP levels rise rapidly during commonly occurring inflammation responses which may confound the results (Young et al. 1991). The influence of inflammation on the performance of the glycan biomarker for HNF1A-MODY is possible yet still less significant than in the case of CRP. Altogether, the promising performance of monitoring plasma α1-3,4 fucosylation as a biomarker for HNF1A-MODY implies that employing easily accessible technology, such as the described exoglycosidase plate-based assay, could serve as a screening step to identify high risk cases and select individuals for the *HNF1A* sequencing which is the current diagnostic gold standard test. This is likely to be cost-saving by narrowing the number of cases requiring expensive genetic testing.

The sequences of *FUT3*, *FUT5* and *FUT6* are highly polymorphic and the presence of single-nucleotide polymorphisms (SNPs) in genes encoding fucosyltransferases might affect α1-3,4 fucosylation levels measured by the plate-based assay (De Vries et al. 2001). Genetic variations can lead to the inactivation of fucosyltransferases, for example, inactive FUT3 in individuals with Lewis negative blood type (King et al. 2019). Information on the presence of SNPs in genes encoding fucosyltransferases was not available in the clinical data of the sample cohort used in this study. Therefore, we are not able to estimate the significance of occurrence of these SNPs on α1-3,4 fucosylation levels measured by the plate-based assay. Exclusion of individuals with loss of function SNPs in fucosyltransferase genes might lead to further improvement of the AUC and different cut-off values for α1-3,4 fucosylation biomarker. We consider this aspect as a limitation of the current study with a potential for future research and investigation.

There are several advantages to the exoglycosidase plate-based assay over other methods used for glycosylation analysis. First, the exoglycosidase plate-based assay allows timely and cost-effective high-throughput screening of large numbers of plasma samples. Currently, the LC-based approach requires extensive sample preparations and a long chromatographic separation of fluorescently tagged glycans, often exceeding 1 h per sample. In comparison, our exoglycosidase plate-based assay provides semiautomated sample preparation using a robotic platform followed by readouts of fluorescence signals that allow the assessment of absolute α1-3,4 fucosylation levels from 96 plasma samples within 24 h. Furthermore, the simple fluorescence readouts generate results that are easy to process and interpret, with no need for high-end instrumentation or expertise. This type of exoglycosidase assay has a potential to become an alternative to immuno-/lectin-based biochemical assays, the use of which might be limited by the availability and binding affinity of the detection antibodies and lectins (Haab 2012). It is expected that advances in glycosidase discovery will enhance a broader application of the exoglycosidase plate-based assay and enable the measurement of absolute levels of other monosaccharides in various types of samples, including released glycans, intact proteins, biopharmaceuticals and clinical samples (Wu et al. 2020).

In conclusion, the exoglycosidase plate-based assay developed in this work enables robust and high-throughput screening of absolute α1-3,4 fucosylation levels in blood plasma samples. The cohort study confirmed the excellent performance of α1-3,4 fucosylation levels as a clinical biomarker for HNF1A-MODY, which allows identification and classification of cases with diabetes carrying damaging variants in the *HNF1A* gene. The results of this work should facilitate the translation of this glycan biomarker into clinical practice and the development of a clinically relevant, widely available diagnostic test.

## Material and methods

### Material

The blood samples were obtained from the HNF1A-MODY cohort (Juszczak et al. 2019). Briefly, participants of this study were recruited from two European centres via the Young Diabetes in Oxford study in the United Kingdom (*n* = 499) and the Croatian National Diabetes Registry (CroDiab) in Croatia (*n* = 501). The study inclusion criteria were the age of 18 years or older and diabetes diagnosis before the age of 45 years. Biochemical inclusion criteria were: fasting C-peptide ≥0.2 nmol/L, which indicates endogenous insulin production and negative GAD antibodies (GADA) to exclude type 1 diabetes patients. Informed consent was obtained from all participants. Sequencing of *HNF1A* and systematic and functional assessment of rare *HNF1A* alleles, performed as part of the previous study (Juszczak et al. 2019), allowed to divide the participants into four groups with (likely) damaging *HNF1A* variants, (likely) benign *HNF1A* variants, a group of cases with VUS and a group without variants in the *HNF1A* gene.

### α1-3,4 fucosylation level measurements

Patients’ blood plasma samples and pooled blood plasma standard (FRNCP0125, VisuCon) were applied in the exoglycosidase plate-based assay. The pooled plasma standard was applied in the assay optimization experiments and used as a process control in the patients’ cohort study. Blood plasma samples were thawed, vortexed and centrifuged briefly at 600 rpm for 30 s prior to the exoglycosidase plate-based assay. Duplicate 10 μL aliquots of each sample were transferred manually into a 96-well PCR plate (4ti-0960, 4titude) and diluted 5-fold in 50-mM citrate buffer, pH 6. The plate was then sealed with a pierce foil seal (4ti-0531, 4titude, Dorking, UK), incubated at 100°C for 10 min to enhance denaturation of proteins, then cooled down at 4°C and centrifuged briefly at 600 rpm for 30 s.

The pierce foil seal was removed before the plate was placed on a Hamilton STARlet liquid handling robot where the next steps were performed using a semiautomated program. The blood plasma samples were treated with an exoglycosidase for fucose release or left untreated (no exoglycosidase). Briefly, 11 μL, α1-3,4-specific fucosidase (E1_10125) (Wu et al. 2020) was added at 3-μM final enzyme concentration in 250-mM citrate buffer, pH 6. The dilution buffer (11 μL) was added to the untreated samples. The plate was then removed from the robot, sealed again, mixed on a plate shaker for 1 min and centrifuged at 800 rpm for 30 s. Following incubation at 37°C overnight (16 ± 1 h), the plate was cooled down at 4°C and briefly centrifuged at 600 rpm for 30 s, the seal removed and the plate was placed back in the robot where 54 μL of ultrapure water was added to each well. The plate was sealed; the samples were mixed by vortexing for 1 min and then centrifuged at 1400 rpm for 40 min.

The seal was then removed and the plate placed into the robot which transferred 54.4 μL of supernatant into a 384-well microplate (4ti-0234, 4titude). An L-fucose assay kit containing reaction buffer, NADP+, fucose dehydrogenase and fucose standard (K-FUCOSE, Megazyme, Bray, Ireland), diaphorase (D5540, Sigma, Poole, UK) and resazurin (199303, Sigma Aldrich, UK) was used in the following steps. A standard curve was prepared in 4 replicates in the 384-well microplate with L-fucose concentration ranging from 0, 0.86, 1.69, 2.52, 3.35, 4.17 to 5.00 ng/μL. A reagent mix (containing 10.9 μL of reaction buffer, pH 9.5, 5.4 μL of 1-mM resazurin solution and 2.7 μL of NADP+) and an enzymatic reagent mix (containing 1.1 μL of fucose dehydrogenase and 5.4 μL of 10 U/mL diaphorase solution) were added to each sample. The plate was then removed from the robot, sealed and incubated for 3 h in the dark at 21°C. The plate was then centrifuged at 600 rpm for 30 s and placed in a plate reader (Enspire 2300, Perkin Elmer Enspire) for fluorescence measurements at 24°C with an excitation wavelength (*λ*_ex_) = 571 nm and an emission wavelength (*λ*_em_) = 586 nm. Three consecutive measurements were taken with excitation illumination from above the plate at a height of 10 mm. Fluorescence signals from the three measurements were averaged.

### Data processing and statistical analysis

Data processing, visualization and statistical analysis were performed using Microsoft Excel and R platform version 1.1.463. ROC curve analysis was applied to estimate diagnostic performance, testing three *HNF1A* variant groups: (likely) damaging, (likely) benign and diabetes cases without *HNF1A* variants. ROC curves, AUC, optimal sensitivity, specificity and cut-off values were generated using “cutpointr” R package. Data outliers were identified with the 1.5×IQR rule and detected per *HNF1A* variant group (Kwak and Kim 2017). Statistical significance of differences in α1-3,4 fucosylation levels between *HNF1A* variant groups was determined using the Mann–Whitney U test for pairwise comparison and Kruskal–Wallis test for global comparison (*P* < 0.05). The Spearman’s correlation method was used to test α1-3,4 fucosylation levels against a panel of inflammatory and metabolic markers available within the cohort. BMI and CRP level values were available in the clinical data for 866 and 916 patients, respectively. The Spearman’s correlation method was used to evaluate α1-3,4 fucosylation levels of glycoconjugates in blood plasma and antennary fucosylation levels of plasma *N*-glycans measured as indexes as a part of the previous HNF1A-MODY study (Demus et al. 2021). A generalized linear model adjusted for either age or sex and high-sensitivity CRP (hsCRP) levels as confounding variables was used to investigate associations between α1-3,4 fucosylation levels (dependent variable) and sex or age.

Chromeleon version 7.2 (Thermo Fisher Scientific, Loughborough, UK) was used to export fluorescence traces as an open text format. UHPLC data processing, quantitation and graphical overlays of chromatograms were performed using HappyTools version 0.0.2 build 1800521a (Jansen et al. 2018). Microsoft Excel was used to export fluorescence readout generated by the microplate reader EnSpire (Perkin Elmer Enspire, Beaconsfield, UK).

For intraplate variation, α1-3,4 fucosylation levels (pg/μL) were averaged for 6 replicates of human blood plasma standards (VisuCon), and then, standard deviation (SD) and coefficient of variation (CV) values were calculated. The averaged α1-3,4 fucosylation levels per each assay plate were used to estimate interplate variation, as described by SD and CV values.

## Authors’ contributions

D.D. carried out all experiments, analyzed and interpreted the study data. P.A.U performed initial development of a prototype for glycosidase plate-based assays. R.A.G supported development and automation of the assay. H.W. and N.J. provided the E1_10125 fucosidase. A.J. performed *HNF1A* sequencing. K.R.O. provided the Oxford study sample cohort and worked on the original biomarker hypothesis. O.G., T.Š. and E.P.M. provided the Croatian study sample cohort. All authors critically reviewed the manuscript and contributed important intellectual content. D.I.R.S. is the guarantor of this work and, as such, had full access to all the data in the study and takes responsibility for the integrity of the data and the accuracy of the data analysis. A.J. was a Diabetes UK George Alberti fellow during the research.

## Supplementary Material

20210928_Supplementary_Information_Demus_et_al_GlycobiologyJ_cwab107

## Data Availability

The datasets generated for this study are available on request to the corresponding author.

## Abbreviations

BMI, body mass index; CRP, C-reactive protein; CV, coefficient of variation; GDP-fucose, guanosine diphosphate-fucose; HNF1A-MODY, Maturity onset diabetes of the young due to damaging alleles in hepatocyte nuclear factor-1 alpha; hsCRP, high-sensitivity CRP; LC, liquid chromatography; NAPD, nicotinamide adenine dinucleotide phosphate; ROC, Receiver Operating Characteristic; SD, standard deviation; SNP, single-nucleotide polymorphism
