# Supplementary material for: Development of an exoglycosidase plate-based assay for detecting α1-3,4 fucosylation biomarker in individuals with HNF1A-MODY
Source: Glycobiology. 2021 Oct 25;32(3):230–8. doi: 10.1093/glycob/cwab107 (PMC8966479; doi:10.1093/glycob/cwab107)
Supplement: 20210928_Supplementary_Information_Demus_et_al_GlycobiologyJ_cwab107 [file 20210928_supplementary_information_demus_et_al_glycobiologyj_cwab107.docx]

**Supplementary Information**

# Development of an exoglycosidase plate-based assay for detecting α1-3,4 fucosylation biomarker in individuals with HNF1A-MODY

Daniel Demus^1,2^, Paulina A. Urbanowicz^1^, Richard A. Gardner^1^, Haiyang Wu^3^, Agata Juszczak^4^, Tamara Štambuk^5,6^, Edita Pape Medvidović^7^, Katharine R. Owen^4,8^, Olga Gornik^6^, Nathalie Juge^3^, Daniel I. R. Spencer^1^

**1** Ludger Ltd, Culham Science Centre, Abingdon, Oxfordshire, England, United Kingdom

**2** Center for Proteomics and Metabolomics, Leiden University Medical Center, Leiden, The Netherlands

**3** Quadram Institute Bioscience, Norwich Research Park, United Kingdom

**4** Oxford Centre for Diabetes, Endocrinology and Metabolism, University of Oxford, Oxford, Oxfordshire, England, United Kingdom

**5** Genos Glycoscience Research Laboratory, Zagreb, Croatia

**6** Faculty of Pharmacy and Biochemistry, University of Zagreb, Zagreb, Croatia

**7** Vuk Vrhovac University Clinic for Diabetes, Endocrinology and Metabolic Diseases, Merkur University Hospital, Zagreb University School of Medicine, Zagreb, Croatia School of Medicine, Zagreb, Croatia

**8** Oxford NIHR Biomedical Research Centre, Oxford Hospitals NHS Foundation Trust, Oxford, Oxfordshire, England, United Kingdom

**Contents**

[Development of an exoglycosidase plate-based assay for detecting α1-3,4 fucosylation biomarker in individuals with HNF1A-MODY 1](#_Toc82099034)

[Table S1. Detection of very low fucose levels in blood plasma samples might be limited by the sample background fluorescence signal. 3](#_Toc82099035)

[Figure S1. Performance of E1_10125 fucosidase applied at different concentrations to 10 µL plasma standard samples 4](#_Toc82099036)

[Figure S2. The efficiency of E1_10125 fucosidase in removing antennary fucose residues from released *N*-glycans and intact glycoproteins present in blood plasma samples. 6](#_Toc82099037)

[Figure S3. Correlations between CRP and α1-3,4 fucosylation levels (A), and BMI and α1-3,4 fucosylation levels (B) observed within the sample cohort 7](#_Toc82099038)

[References 8](#_Toc82099039)

Table S1. Detection of very low fucose levels in blood plasma samples might be limited by the sample background fluorescence signal. The table presents a summary of an experiment using 3 sets of plasma samples spiked with fucose standard of known concentration (in a range from 1200.00 to 0.04 pg/µL). The fucose-spiked denatured plasma samples were not exoglycosidase treated and were processed by the plate-based assay: the samples were centrifuged, the supernatants were subjected to redox reactions and the fluorescence signal measurements. Data are expressed as the mean and standard deviation (SD) of fluorescence signal together with coefficient of variation (CV) and signal to background (S/B) ratio for each concentration. S/B values below 0 (marked in red) indicate concentrations of fucose in plasma samples that were below limit of detection of the assay.

| Fucose concentration in spiked plasma samples (pg/µL) | Average signal - S (U), n = 3 | SD | CV | Background signal - B (U) | Average background signal – B_A_ (U) | S/B_A_ |
| --- | --- | --- | --- | --- | --- | --- |
| 1200.00 | 104152 | 1309 | 1% | 34374 | 34833 | 2.99 |
| 600.00 | 71160 | 1596 | 2% | 32480 |  | 2.04 |
| 300.00 | 51872 | 856 | 2% | 35263 |  | 1.49 |
| 150.00 | 40864 | 1074 | 3% | 34817 |  | 1.17 |
| 75.00 | 38249 | 1019 | 3% | 34653 |  | 1.10 |
| 37.50 | 35735 | 1746 | 5% | 34190 |  | 1.03 |
| 18.75 | 34320 | 1697 | 5% | 32224 |  | 0.99 |
| 9.38 | 32343 | 44 | 0% | 32193 |  | 0.93 |
| 4.69 | 34245 | 1360 | 4% | 39285 |  | 0.98 |
| 2.34 | 33753 | 1639 | 5% | 35227 |  | 0.97 |
| 1.17 | 35390 | 179 | 1% | 37565 |  | 1.02 |
| 0.59 | 34515 | 1292 | 4% | 35536 |  | 0.99 |
| 0.29 | 33617 | 1605 | 5% | 34514 |  | 0.97 |
| 0.15 | 34145 | 899 | 3% | 35346 |  | 0.98 |
| 0.07 | 33578 | 1613 | 5% | 32183 |  | 0.96 |
| 0.04 | 34112 | 227 | 1% | 32312 |  | 0.98 |


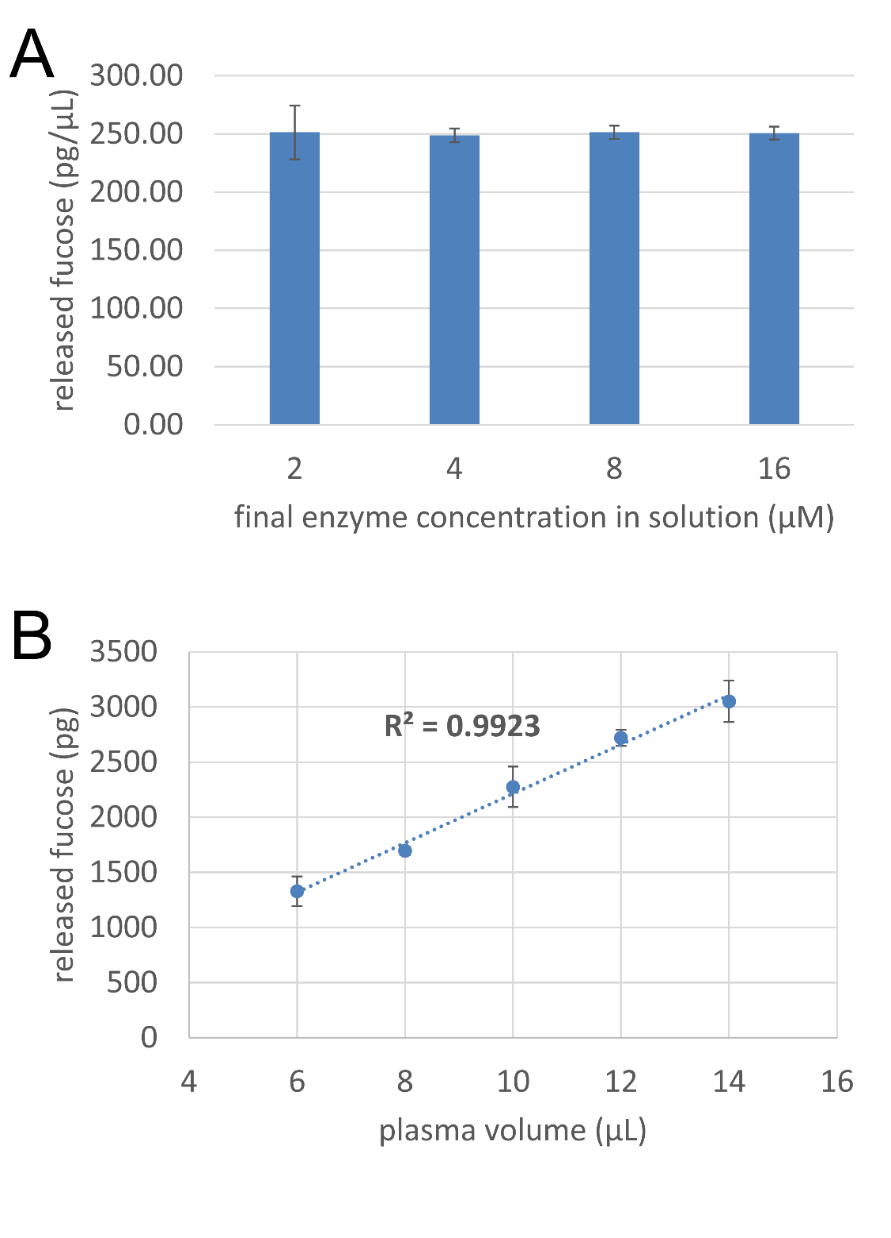


Figure S1. Performance of E1_10125 fucosidase applied at different concentrations to 10 µL plasma standard samples, n = 3 (A) and E1_10125 fucosidase applied at the final concentration of 3 µM to a range of plasma volumes, n = 3 each (B). E1_10125 provides maximum fucose release from blood plasma conjugates at the range of 2 – 16 µM final concentrations (A). 3 µM final concentration was proven to provide a good linearity (R^2^ = 0.9923) for the released fucose amounts within the tested plasma volumes that mimicked the protein content variation in 10 µL of clinical blood plasma samples.


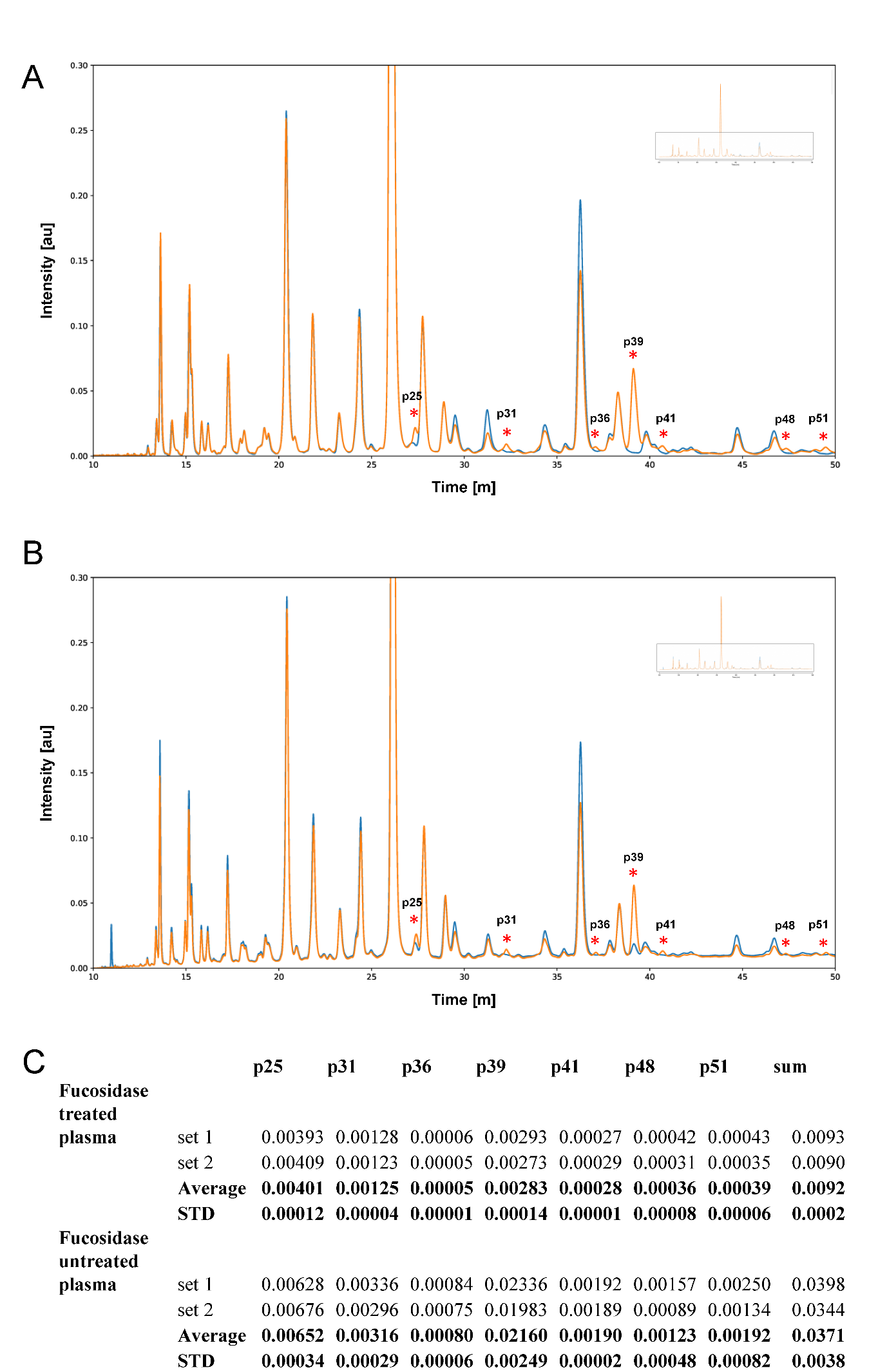


Figure S2. The efficiency of E1_10125 fucosidase in removing antennary fucose residues from released *N*-glycans and intact glycoproteins present in blood plasma samples. Fluorescence UHPLC chromatograms of released procainamide labeled plasma *N*-glycans derived from 8 µL pooled plasma standard (A) and plasma pellet obtained from the plate assay (B), and a table showing relative abundance of α1-3,4 fucosylated *N*-glycan peaks in full plasma *N*-glycome obtained from the plasma pellets (C). E1_10125 fucosidase digested samples (blue) were overlaid with enzymatically untreated sample chromatograms (orange). Chromatograms were normalised to the most abundant peak. α1-3,4 fucosylated glycan structure peaks are marked with asterisks (*). *N*-glycan release, exoglycosidase digestions, chromatographic separation of *N*-glycan structures and relative peak area extraction were performed as described previously (Demus et al., 2021; Kozak et al., 2015), and the content of antennary fucosylated *N*-glycans calculated for both sets of samples, each set n =2. Briefly, two sets (n = 2) of blood plasma pellets from 10 µL plasma standard (VisuCon) from a set of exoglycosidase treated and untreated samples for the plate-based assay were saved for *N*-glycan release to assess a degree of fucose release performed on denatured intact glycoproteins. Additionally, 8 µL pooled plasma standard (n = 2 sets) was used for *N*-glycan release and digestion by E1_10125 as positive control. Both sets were then compared for the degree of fucose release from intact glycoproteins vs. released *N*-glycans. The results showed that E1_10125 allows full (100%) α1-3,4 linked fucose release from released *N*-glycans whereas E1_10125 at the final concentration of 3 µM allows approximately 75% fucose release from *N*-glycans of denatured intact glycoproteins, which was calculated based on relative abundance of α1-3,4 fucosylated *N*-glycan peaks in fucosidase treated (0.0092 ± 0.0002) and untreated (0.0371 ± 0.0038) plasma samples (C).


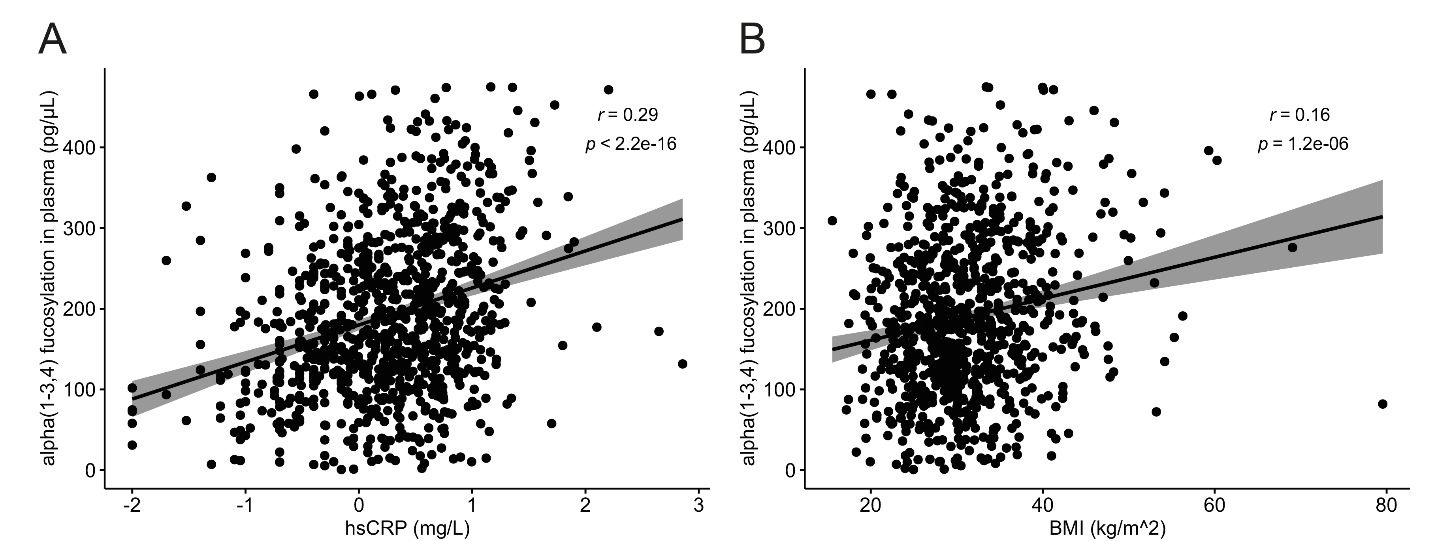


Figure S3. Correlations between CRP and α1-3,4 fucosylation levels (A), and BMI and α1-3,4 fucosylation levels (B) observed within the sample cohort. hsCRP concentrations were logarithmically transformed.

# References

Demus, D., Jansen, B. C., Gardner, R. A., Urbanowicz, P. A., Wu, H., Štambuk, T., Juszczak, A., Medvidović, E. P., Juge, N., Gornik, O., Owen, K. R., & Spencer, D. I. R. (2021). Interlaboratory evaluation of plasma N-glycan antennary fucosylation as a clinical biomarker for HNF1A-MODY using liquid chromatography methods. *Glycoconjugate Journal*. https://doi.org/10.1007/s10719-021-09992-w

Kozak, R. P., Tortosa, C. B., Fernandes, D. L., & Spencer, D. I. R. (2015). Comparison of procainamide and 2-aminobenzamide labeling for profiling and identification of glycans liquid chromatography with fluorescence detection coupled to electrospray ionization-mass spectrometry. *Analytical Biochemistry*, *486*, 38–40. https://doi.org/10.1016/j.ab.2015.06.006
